# Cardiac vagal activity is associated with gut-microbiome patterns in women—An exploratory pilot study

**DOI:** 10.1080/19585969.2022.2128697

**Published:** 2022-10-11

**Authors:** Sabrina Mörkl, Andreas Oberascher, Josef M. Tatschl, Sonja Lackner, Thomaz F. S. Bastiaanssen, Mary I. Butler, Maximilian Moser, Matthias Frühwirth, Harald Mangge, John F. Cryan, Timothy G. Dinan, Sandra J. Holasek

**Affiliations:** a Department of Psychiatry and Psychotherapeutic Medicine, Medical University of Graz, Graz, Austria; b Department of Psychiatry, Psychotherapy and Psychosomatics, Paracelsus Medical University of Salzburg, Salzburg, Austria; c Division of Physiology, Otto Loewi Research Center (for Vascular Biology, Immunology and Inflammation), Medical University of Graz, Graz, Austria; d Health Psychology Unit, University of Graz, Graz, Austria; e Division of Immunology and Pathophysiology, Otto Loewi Research Center (for Vascular Biology, Immunology and Inflammation), Medical University of Graz, Graz, Austria; f APC Microbiome Ireland, University College Cork, Cork, Ireland; g Department of Psychiatry and Clinical Neuroscience, University College Cork, Cork, Ireland; h Human Research Institute of Health Technology and Prevention Research, Weiz, Austria; i Clinical Institute of Medical and Chemical Laboratory Diagnostics, Medical University of Graz, Graz, Austria

**Keywords:** Gut microbiota, vagal nerve, gut-brain-axis, autonomic nervous system, heart rate variability

## Abstract

**Introduction:**

A functional reciprocity between the gut microbiome and vagal nerve activity has been suggested, however, human studies addressing this phenomenon are limited.

**Methods:**

Twenty-four-hour cardiac vagal activity (CVA) was assessed from 73 female participants (aged 24.5 ± 4.3 years). Additionally, stool samples were subjected to 16SrRNA gene analysis (V1–V2). Quantitative Insights Into Microbial Ecology (QIIME) was used to analyse microbiome data. Additionally, inflammatory parameters (such as CRP and IL-6) were derived from serum samples.

**Results:**

Daytime CVA correlated significantly with gut microbiota diversity (*r*_sp_ = 0.254, *p* = 0.030), CRP (*r*_sp_ = −0.348, *p* = 0.003), and IL-6 (*r*_sp_ = −0.320, *p* = 0.006). When the group was divided at the median of 24 h CVA (Mdn = 1.322), the following features were more abundant in the high CVA group: *Clostridia* (Linear discriminant analysis effect size (LDA) = 4.195, *p* = 0.029), *Clostridiales* (LDA = 4.195, *p* = 0.029), *Lachnospira* (LDA = 3.489, *p* = 0.004), *Ruminococcaceae* (LDA = 4.073, *p* = 0.010), *Faecalibacterium* (LDA = 3.982, *p* = 0.042), *Lactobacillales* (LDA = 3.317, *p* = 0.029), *Bacilli* (LDA = 3.294, *p* = 0.0350), *Streptococcaceae* (LDA = 3.353, *p* = 0.006), *Streptococcus* (LDA = 3.332, *p* = 0.011). Based on Dirichlet multinomial mixtures two enterotypes could be detected, which differed significantly in CVA, age, BMI, CRP, IL-6, and diversity.

**Conclusions:**

As an indicator of gut-brain communication, gut microbiome analysis could be extended by measurements of CVA to enhance our understanding of signalling *via* microbiota-gut-brain-axis and its alterations through psychobiotics.

## Introduction

The autonomic nervous system (ANS) is an important adaptor to the external and internal environment. This complex control system and its circadian oscillation are crucial to maintaining the homeodynamic equilibrium of the body. Compromised ANS functioning has been linked to a range of mental and physical disorders, such as depression (Koch et al. 2019). The vagal nerve (VN) derives its name from the Latin for ‘*wandering*’, due to its ubiquitous innervation of the visceral organs (Berthoud and Neuhuber 2000). Continuously, the VN, which consists of 80% afferent and 20% efferent fibres (Bonaz et al. 2018), acts as an essential bidirectional communication pathway to present information to and from the cardiovascular system, the respiratory system, and the gastrointestinal tract (Fulling et al. 2019; O'Connor et al. 2019).

Over the past decade, there has been increasing emphasis on the relationship between the trillions of bacteria in the gut (the microbiota) and brain function (Bastiaanssen et al. 2019; Sarkar et al. 2018). The VN afferents recognise gut microbiota and their metabolites, convey information to the central nervous system (Bravo et al. 2011; Bonaz et al. 2018), and therefore are an essential communication pathway of the microbiota-gut-brain-axis (MGBA). An altered faecal microbiota together with altered microbial diversity has been identified in many psychiatric disorders, such as affective disorders (Jiang et al. 2015; Kelly et al. 2016) and anorexia nervosa (Mörkl et al. 2017).

A validated marker of ANS functioning is heart rate variability (HRV), which describes the variation in the time intervals between adjacent heartbeats (Shaffer and Ginsberg 2017). Importantly, HRV is sensitive to cardiac vagal activity (CVA), thus allowing the non-invasive assessment of the latter using electrocardiography. Considering the role of the VN as an important link in gut-brain communication, assessing HRV in conjunction with the microbiota could provide a feasible tool to clinically explore these interactions (Bonaz et al. 2016, 2018).

Although the gut microbiome is linked to the function of the VN, data on the link between vagal nerve function and gut microbiota in humans are limited. To our knowledge, there has been no study to date investigating gut microbiota and 24-h CVA.

Only one study has investigated the interdependency between the VN and microbiota composition using short-term HRV (i.e., 10 min) in children, finding that higher CVA was associated with higher alpha diversity (Michels et al. 2019). However, since CVA shows a circadian rhythm, 24-h measurements could provide more precise insights into microbiota-brain communication (Valladares et al. 2008). Moreover, HRV and microbiota patterns seem to be age-dependent and change throughout the lifespan (Umetani et al. 1998; Lehofer et al. 1999; O'Toole and Claesson 2010). Additionally, both, gut microbiota and vagal nerve function seem to be closely interconnected with inflammation (Pavlov and Tracey 2012; Soares-Miranda et al. 2012; Al Bander et al. 2020). Therefore, the present work set out to expand current research by evaluating for the first time, microbiota composition and 24 h CVA measurements in female adults.

The objectives of this study were: (1) to determine if there is a relationship between CVA and diversity of the gut microbiome, (2) to investigate whether CVA correlates with parameters of inflammation (CRP, IL-6) and depression, (3) to investigate whether CVA differs with regard to enterotype, (4) to examine whether high and low CVA can be expressed as distinct gut microbiome patterns.

## Methods

### Participants

Seventy-three participants of the ESAN-project (Mörkl et al. 2017) were included in the study. Participants were recruited at the university campus and hospitals in Graz. Study participants met the inclusion criteria for the ESAN study published elsewhere (Mörkl et al. 2017). This study was conducted according to the Declaration of Helsinki and was part of the ESAN-project, which was approved by the ethics committee of the Medical University of Graz (MUG-26-383ex13/14). Every participant provided written informed consent.

### CVA

R-R intervals (RRI) were assessed using a single-channel high-precision ECG monitor (ChronoCord^®^, 7th generation, Human Research Institute, Weiz, Austria, 8000 samples/s, 16 bit) (Moser et al. 1994, 2008). The ChronoCord^®^ is a miniaturised ECG recorder that allows subjects to engage in normal daily activities. Three adhesive electrodes were placed on the participants’ trunk (sternum, 5th left intercostal space, and on the right side of the trunk between the 11th and 12th rib). The ChronoCord^®^ was attached to the waistband of the subject, recording for 24 h, yielding ∼110,000 RRIs. Data were stored for further software evaluation (Chronobase^®^, Human Research Institut, Weiz, Austria; https://www.humanreasearch.at). The subjects were instructed to take note of the time of light off in the evening and awakening in the morning. The RRI time series was filtered, and artefacts were removed according to Grote et al. (2021). R peaks were detected by a digital filter described in Moser et al. (1994) and Lehofer et al. (1999) to more than 1 ms accuracy, adhering to task force guidelines (Rawenwaaij-Arts et al. 1996).

As a marker of CVA, the respiratory sinus arrhythmia (RSA) was extracted from the RRI time series, using a time-domain method according to Moser et al. (1994), which has been shown to yield a robust estimate of cardiorespiratory interactions (Moser et al. 1994; Topçu et al. 2018). The RSA describes the respiratory-driven fluctuations of the heart rate and is primarily mediated by vagal nerve activity (Schwerdtfeger et al. 2020). For a detailed description of the specific RSA assessment used in our study please see the work of Moser et al. (1994) and Topçu et al. (2018). Logarithmic transformation was conducted for the RSA (logRSA) when normality was violated (Laborde et al. 2017). CVA was analysed for 24-h, wake and sleep phases, respectively.

### Questionnaires

We surveyed demographical and clinical data (age, weight, height). Participants completed the Beck Depression Inventory (BDI) (Beck et al. 1961) and the Hamilton Depression Rating Scale (HAM-D) (Hamilton 1960).

### Inflammatory parameters

C-Reactive Protein (CRP) was measured by a particle-enhanced turbidimetric assay (Cobas 8000 analyser, module c 701, Roche Diagnostics, Mannheim, Germany). The limit of quantification for CRP was 0.2 mg/L. The intra-assay and inter-assay coefficients of variation of assays were below 5%. Interleukin-6 (IL-6) was determined with an ElectroChemiLuminescence ImmunoAssay (ECLIA) (Cobas 8000 analyser, module e 801, Roche Diagnostics, Mannheim, Germany).

### Gut microbiome analysis

The methods of microbiome analysis have been described in detail elsewhere (Mörkl et al. 2017). The following paragraphs give a brief overview. Stool samples were collected with the PSP spin stool DNA stool collection kit (Stratec, Birkenfeld, Germany). Approximately 1 g of the sample was suspended in the PSP-Spin-Stool-DNA-Plus-Kit-buffer-solution. All samples were stored in a −20 °C-freezer. Bacterial DNA from stool samples was extracted using the PowerLyzer PowerSoil DNA Isolation Kit (MO BIO Laboratories Inc, CA, USA). DNA concentration was measured by Picogreen-fluorescence (Thermo Fisher Scientific). The variable V1–V2 region of the bacterial 16S rRNA gene was amplified with Polymerase-chain-reaction (PCR) (oligonucleotide primers 515f:GATTGCCAGCAGCCGCGGTAA and 806r:GGACTACCAGGGTATCTAAT). Bacterial 16S rRNA was amplified with the Mastermix 16S Complete PCR Kit (Molzym, Bremen, Germany). The first PCR reaction product was subjected to a second round of PCR with primers fusing the 16S primer sequence to the adapters for Ion-Torrent-sequencing. PCR products were subjected to agarose gel electrophoresis. The band of the expected length (about 330 nt) was excised and purified (QiaQick gel extraction system; Qiagen, Hilden, Germany). DNA concentration was measured with Picogreen-fluorescence. Amplicons were pooled equimolarly and subjected to PCR. The beads were purified on an Ion ES station and loaded onto Ion Torrent 318 chips. Sequencing reactions were performed on an Ion Torrent PGM using the Ion 400BP Sequencing Kit (all reagents were from Thermo Fisher Scientific, MA, USA). Sequences were split by barcode and transferred to the Torrent suite server. Unmapped bam files were used as input for bioinformatics.

### Analysis of microbiome data

Sequences were assessed with the FASTQ tool. Paired-end reads were pre-filtered (using the quality threshold of >28), trimmed, and filtered for quality and chimaeras using the DADA2 library in R (Callahan et al. 2016). DADA2 was used to assign taxonomy against the SILVA SSURef database (release v132) (Quast et al. 2013) with the recommended parameters stated in the DADA2 manual. Operational taxonomic units (OTUs) that were unknown on the genus level were not considered in downstream analysis, as were OTUs that were only detected as non-zero in ten percent or fewer of total samples. The diversity of bacterial taxa was estimated with Chao-1 (Chao 1984). Linear discriminant analysis Effect Size (LEfSe) (Segata et al. 2011) was used to identify differentially abundant taxa with Quantitative Insights Into Microbial Ecology (QIIME)-scripts (Caporaso et al. 2010) using default settings on the galaxy-server of the Medical University of Graz (*galaxy.medunigraz.at*). Enterotype distribution (using Dirichlet multinomial mixtures) was assessed with R (Version 3.6) (Holmes et al. 2012). Principal component analysis was performed on centre log-ratio (clr) transformed data using the ALDEx2 library in R (Fernandes et al. 2013).

### Statistical analysis and visualisation

All data are presented as mean and standard deviation unless otherwise specified. Depending on the distribution of data, to identify differences between groups we performed either an ANOVA or a Kruskal–Wallis test and a Mann–Whitney *U* test. Analyses were conducted in SPSS Version 23.0 (IBM Corp. IBM SPSS Statistics for Windows, Version 23.0., IBM Corp., Armonk, NY, USA). Data visualisation was performed using QIIME-outputs (Caporaso et al. 2010). All tests were two-tailed, with *p* < 0.05 considered significant.

## Results

### Demographical and clinical characteristics

Seventy-three female participants from the ESAN study (*n* = 12 patients with anorexia nervosa, 14 normal-weight participants, 16 overweight participants, 13 participants with grade-1 obesity, and 18 normal-weight athletes) provided HRV data for this project. The demographics and clinical characteristics are shown in Table 1.

**Table 1.** Characteristics of the study participants.

Population characteristics	Mdn (IQR)
Age (years)	24.00 (6.25)
BMI (kg m^−2^)	23.32 (6.75)
BDI-Score	5.00 (10.25)
HAM-D-Score	4.00 (8.00)
Interleukine-6 (pg/mL)	1.90 (1.82)
CRP (mg/L)	1.45 (3.62)

BMI: body mass index; BDI: Beck depression inventory; HAM-D: Hamilton rating scale for depression; CRP: C-reactive protein. Data are presented as median (Mdn) and interquartile range (IQR).

### Correlations of CVA and gut microbiota diversity

Gut microbiota diversity (Chao-1 index) was correlated using Spearman’s correlations.

Chao-1-diversity index correlated positively with daytime CVA (*r*_sp_ = 0.254, *p* = 0.030).

### Correlations of CVA and inflammation (CRP, IL-6)

CRP correlated significantly with 24-h CVA (*r*_sp_ = −0.391, *p* = 0.001), daytime CVA (*r*_sp_ = −0.348, *p* = 0.003) and night-time CVA (*r*_sp_ = −0.350, *p* = 0.002). IL-6 correlated significantly with 24-h CVA (*r*_sp_ = −0.440, *p* < 0.001), daytime CVA (*r*_sp_ = −0.320, *p* = 0.006), and night-time CVA (*r*_sp_ = −0.440, *p* < 0.001).

### Correlations of CVA and depression scores (BDI, HAMD)

BDI correlated with 24-h CVA (*r*_sp_ = −0.273, *p* = 0.022). and daytime CVA (*r*_sp_ = −0.309, *p* = 0.009). HAMD showed significant correlations with daytime CVA (*r*_sp_ = −0.239, *p* = 0.043).

### LEfSe-analysis

When the group was divided using the median of 24-h CVA (Mdn = 1.322), the following features were more abundant in the high CVA group and therefore more prevalent in participants with higher vagal function: *Clostridia* (LDA = 4.195, *p* = 0.029), *Clostridiales* (LDA = 4.195, *p* = 0.029), *Lachnospira* (LDA = 3.489, *p* = 0.004), *Ruminococcaceae* (LDA = 4.073, *p* = 0.010), *Faecalibacterium* (LDA = 3.982, *p* = 0.042), *Lactobacillales* (LDA = 3.317, *p* = 0.029), *Bacilli* (LDA = 3.294, *p* = 0.0350), *Streptococcaceae* (LDA = 3.353, *p* = 0.006), *Streptococcus* (LDA = 3.332, *p* = 0.011).

*Ruminococcaceae* (LDA = 4.069, *p* = 0.007) were predominantly found in the group with daytime CVA above the median (Mdn = 1.203), while *Eggerthella* (LDA = 2.393, p = 0.006) was predominantly found in the group with low daytime CVA.

When CVA at night-time was divided by the median (Mdn = 1.535) the following bacteria were more abundant in the group with high CVA: *Streptococcus* (LDA = 3.659, *p* = 0.027), *Streptococcaceae* (LDA = 3.673, *p* = 0.016), *Bacteroides* (LDA = 4.302, *p* = 0.018), *Bacteroidaceae* (LDA = 4.312, *p* = 0.018), *Lachnospira* (LDA = 3.519, *p* = 0.006), *Ruminococcaceae* (LDA = 4.051, *p* = 0.048), *Faecalibacterium* (LDA = 4.041, *p* = 0.018).

In the group with low CVA at night-time, the following features were more abundant: *Clostridiaceae* (LDA = 3.220, *p* = 0.037), *Lactobacillus* (LDA = 0.372, *p* = 0.018), *Bifidobacterium* (LDA = 4.157, *p* = 0.048), *Bifidobacteriaceae* (LDA = 4.157, *p* = 0.048), *Bifidobacteriales* (LDA = 4.157, *p* = 0.048), *Actinobacteria* (LDA = 4.166, *p* = 0.047), *Dorea* (LDA = 2.962, *p* = 0.049), and *Oscillospira* (LDA = 3.057, *p* = 0.025).

### Enterotypes

**Figure 1.**
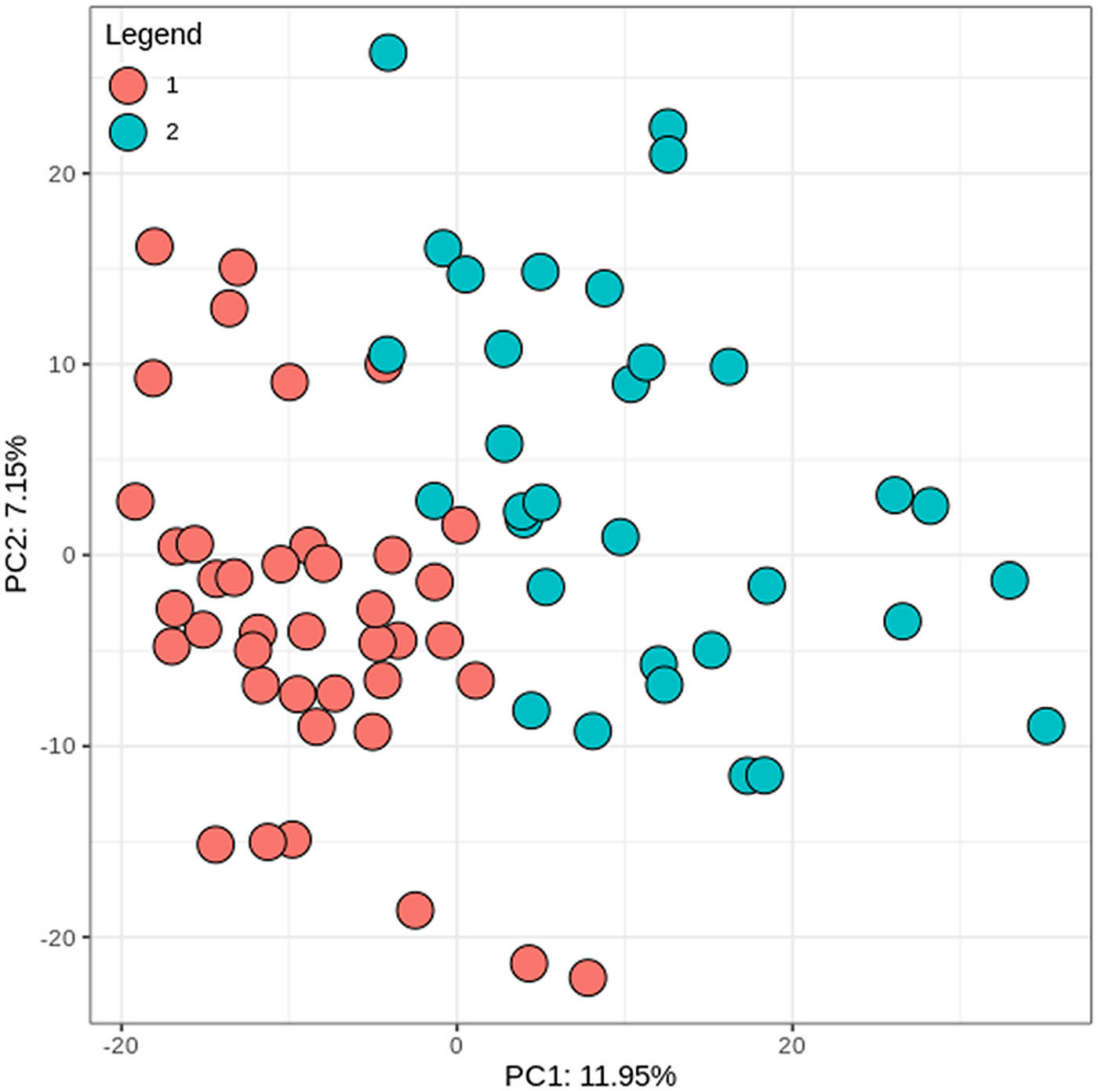
Clustering of study participants in two distinct enterotypes. PC: principal component.

Enterotypes are a classification of a bacteriological ecosystem. Based on multinomial mixtures (Holmes et al. 2012), we could determine two enterotypes of the gut microbiome of the study participants, where 41 subjects belonged to enterotype 1 and 32 subjects belonged to enterotype 2. Figure 1 shows the clustering of the participants allocated to enterotype 1 and enterotype 2.

LEfSe (Segata et al. 2011) was used to determine the predominant taxa in these enterotypes and yielded 37 differentially abundant features. Supplementary Table 2 lists the LDA and *p*-values of differentially abundant bacterial features, among them 18 features had abundances in the percental range. In enterotype 1 the following bacterial groups were more abundant: *Coprococcus, Clostridiales, Rikenellaceae, Barnesiellaceae, Ruminococcaceae, Alphaproteobacteria, Odoribacter, Erysipelotrichaceae, Butyricimonas, Methanobrevibacter, Akkermansia, Coriobacteriaceae.* In enterotype 2, *Bacteroides, Blautia, Dialister, Ruminococcus, Eubacterium,* and *Dorea* were more abundant than in enterotype 1.

Enterotype-groups differed significantly regarding age, *t*(52.91) = −2.392, *p* = 0.020; BMI, *t*(52.1) = −2.792, *p* = 0.007; CRP, *t*(49.27) = −2.657, *p* = 0.011; *d* = 3.85, IL-6, *t*(39.75) = −2.077, *p* = 0.044, gut microbiota diversity measured with Chao-1 diversity index, *t*(71) = 3.934, *p* < 0.001, CVA during daytime, *t*(71) = 2.580, *p* = 0.014 and 24 h CVA *t*(71) = 2.176, *p* = 0.033, whereby participants with enterotype 1 had lower age, lower BMI, lower CRP, lower IL-6, higher gut microbiota diversity and higher vagal function.

## Discussion

In this study, we have shown, for the first time, that long-term CVA was positively correlated with gut microbiota diversity and inversely with inflammatory parameters, such as IL-6 and CRP. Further, we identified specific microbial communities more abundant in participants with higher CVA, such as *Clostridia, Lachnospira, Ruminococaceae, Faecalibacterium, Lactobacillales,* and *Streptococcaceae*. We identified two gut microbial enterotypes which differed significantly in terms of CVA, gut microbiota diversity, age, BMI, CRP, and IL-6.

To our knowledge, this is the first human study to link the gut microbiota with a 24 h assessment of CVA. Only one study, including 93 Belgian children, investigated both the gut microbiota and short-term CVA (i.e., pnn50, the percentage of successive RR intervals that differed by more than 50 ms (Shaffer and Ginsberg 2017) over 5 min selected from 10-min-measurements) (Michels et al. 2019). Although CVA can be retrieved from short-term recordings, long-term HRV measurements provide additional information as for example CVA exhibits circadian fluctuations, thus providing a more comprehensive marker of ANS functioning (Laborde et al. 2017). Hence, an integrated 24 h HRV analysis may be better suited to examine the functional reciprocity between the VN and the gut microbiota.

An additional study including 113 Belgian children (8–16 years) derived short-term CVA (i.e., high frequency from 5-min HRV measurements) without investigating gut microbiota but bacterial metabolites (Michels et al. 2017) and found that higher parasympathetic activity was related to lower valerate levels.

### Alpha diversity

We identified a positive correlation between bacterial alpha diversity measured with Chao-1 and CVA during the daytime. Our findings are consistent with those of the earlier aforementioned study which demonstrated a correlation between short-term vagal activity (pnn50) and alpha diversity in children (Michels et al. 2019), indicating that a higher bacterial diversity corresponds positively with CVA. Greater bacterial diversity is often associated with beneficial health states, although the role of alpha diversity as a general marker for good gut health is debated (Mosca et al. 2016; Kuo and Chung 2019). Nonetheless, the correlation of gut microbiota diversity with diurnal vagal activity warrants further investigation.

### Vagus nerve, inflammation, and depression

As hypothesised, CVA was inversely correlated with inflammatory markers. Previous publications have described a cholinergic anti-inflammatory pathway connecting the vagal system to the immune system (Rosas-Ballina and Tracey 2009; Tracey 2002). Importantly, our findings support a recent meta-analysis finding negative associations between HRV measures with inflammation (Williams et al. 2019). Thus, sufficient VN function could dampen inflammation by directly effecting immune cells as well as by decreasing intestinal permeability (Carabotti et al. 2015). Interestingly, psychiatric conditions, such as depression show altered HRV (Koch et al. 2019) as well as increased inflammation (Valkanova et al. 2013). Noteworthy, depression recovery can be facilitated via stimulating the vagus nerve either electrically or *via* slow-paced deep breathing (Carreno and Frazer 2017; Tatschl et al. 2020). Intriguingly, the amplification of CVA due to slow-paced deep breathing could be enhanced by complementing the latter with inspiratory resistance or pelvic floor recruitment during inhalation (Gholamrezaei et al. 2021; Tatschl and Schwerdtfeger 2022).

### Taxonomic differences

When our study participants were divided according to CVA some members of the phylum *Firmicutes* and the class *Clostridia* (order: *Clostridiales*, family: *Ruminoccoccaceae,* genus: *Lachnospira*), as well as the class *Bacilli* (order: *L*a*ctobacillales*, family*: Streptococcaceae*) were more abundant in the group with higher vagal activity. We did not find any studies to support or contradict our results on gut microbiota and vagal nerve function in adults. However, our study results are in line with the aforementioned study of Michels et al. where low vagal activity as measured by pnn50 was associated with low *Firmicutes* and low *Clostridiales* in children (Michels et al. 2019).

### Enterotypes

Of note, we show for the first time, that a specific enterotype seems to be connected to the function of the vagal nerve. Participants belonging to enterotype 1 had a significantly better vagal function, higher gut microbiota diversity, lower BMI, and lower inflammation (CRP and IL-6). This enterotype contained high abundances of diverse features, such as *Coprococcus, Clostridiales, Rikenellaceae, Barnesiellaceae, Ruminococcaceae, Alphaproteobacteria, Odoribacter, Erysipelotrichaceae, Butyricimonas, Methanobrevibacter, Akkermansia, Coriobacteriaceae* (Supplementary Table 2). Interestingly, in the Flemish gut flora project with over 1000 participants, *Coprococcus* was found to be depleted in depression and together with *Faecalibacterium* was associated with a higher quality of life (Valles-Colomer et al. 2019). Also, *Clostridiales* was found to be a predominant microbial group to mediate psychiatric disorders (Li et al. 2020). Most bacteria in enterotype 1 (e.g., *Clostridiales, Coprococcus, Ruminococcaceae, Akkermansia*) produce short-chain fatty acids (SCFA), such as butyrate, propionate, and acetate, which are important metabolites for maintaining intestinal homeostasis and gut barrier function (Parada Venegas et al. 2019).

### Limitations

Our study has several limitations. First, this study was conducted on female participants only. Results of previous studies have demonstrated sex differences regarding CVA (Valladares et al. 2008; Koenig and Thayer 2016). Further, we did not take potential influences of the menstrual cycle of participants into account as stool samples were collected cross-sectionally. The gut-brain axis is modulated via sex hormones, such as oestrogen (Yoon and Kim 2021); this could also have had an influence on vagal function, and HRV is known to vary with the menstrual cycle as well (Seebauer et al. 2002). Also, both heart rate variability and gut microbiome composition are influenced by BMI and diet (de Lartigue 2016; Daniel 2021). However, due to the limited sample size of this pilot study, their potential moderating effect was not assessed. Hence, subsequent well-powered studies should address this major limitation of this current pilot recruiting larger samples. Notably, the anorexia nervosa patients in our study remained on their treatment as usual antidepressant therapy (most were taking SSRIs and SNRIs in various dosages). Several antidepressants were shown to have effects on gut microbiome composition and HRV measurements (van Zyl et al. 2008; McGovern et al. 2019). Though there is evidence that many antidepressants have antibacterial effects, this evidence is primarily from *in-vitro* and animal studies, and the dosage and substance-dependent impact of each of these medications on the human gut microbiome is still unknown (Bohnert et al. 2011; Ayaz et al. 2015; Younis et al. 2017; Cussotto et al. 2019).

Microbiota diversity was found to be correlated to colonic transit time, which was not assessed in this study (Roager et al. 2016). Another limitation is the cross-sectional study design. Future, longitudinal studies should address how HRV parameters and gut microbiome composition change in the long term and how interventions (e.g., with diet, psychobiotics, or vagal nerve stimulation) affect both vagal activity and the gut microbiome.

## Conclusions

This pilot study indicates that long-term CVA is associated with gut microbiome patterns in women. Hence, integrating HRV assessment in future gut microbiota research offers a feasible and non-invasive approach to generate a more comprehensive assessment of the microbiota-gut-brain axis. Importantly, future studies should investigate how modulating the gut microbiome through psychobiotics, such as dietary interventions and supplements could affect vagal nerve functioning in health and disease (Bonaz et al. 2016; Carreno and Frazer 2017; Wu et al. 2018). Finally, accounting for the functional reciprocity of the gut-brain axis, follow-up studies could also address whether stimulating the VN electrically or via slow-paced deep breathing can modify the microbiome and/or intestinal barrier integrity (Carreno and Frazer 2017; Bonaz et al. 2018).

## Supplementary Material

Supplemental Material

## Data Availability

Data are made available on figshare under the DOI-number: 10.6084/m9.figshare.19615887.
